# Supplementary material for: Baseline Cytokine Profile Identifies a Favorable Outcome in a Subgroup of Colorectal Cancer Patients Treated with Regorafenib
Source: Vaccines (Basel). 2023 Feb 2;11(2):335. doi: 10.3390/vaccines11020335 (PMC9959285; doi:10.3390/vaccines11020335)
Supplement: Supplementary file 1 [file vaccines-11-00335-s001.zip › vaccines-2134689-supplementary.pdf]

Table S1. Metastatic sites.

Cluster 1

Patient's Id	iver	Lung	Lymphnodes	Peritoneum	Bone	Other
REG2	X	X			X	
REG3		X	X	X		
REG6		X		X		X
REG7		X				
REG8		X				
REG9	X		X	X		
REG11	X	X				
REG17	X	X	X			
REG19	X	X	X		X	
REG21	X			X		
REG22	X	X				
REG24		X		X		
REG26	X	X				

Cluster2

Paztient's Id	Liver	Lung	Lymphnodes	Peritoneum	Bone	Other
REG1				X		
REG4	X					
REG5	X					
REG10	X	X	X		X	
REG14	X	X			X	
REG15	X	X	X			
REG16			X		X	X
REG18	X	X	X			
REG20		X		X		
REG23	X					
REG25	X	X	X			

Cluster 3

Patient's Id	Liver	Lung	Lymphnodes	Peritoneum	Bone	Other
REG13	X	X	X	X		X

Table S2.ROC table analysis of variables confronting patients above median PFS compared to patients below median. Larger values of the test result variable(s) indicate stronger evidence for patients below median PFS.

Area Under the Curve

Test Result Variable(s)	Area	Std. Error[a]	Asymptotic Sig.[b]	Asymptotic 95% Confidence Interval	
				Lower Bound	Upper Bound
TGF-β	.795	.098	.012	.602	.988
TNF-α	.744	.113	.039	.522	.965
VEGF	.494	.123	.957	.253	.734
IFN-γ	.558	.118	.624	.327	.788
IL-2	.494	.121	.957	.257	.731
IL-4	.580	.119	.497	.348	.812
IL-5	.519	.121	.870	.282	.757
IL-6	.808	.093	.009	.626	.990
IL-8	.740	.101	.041	.542	.939
IL-10	.766	.095	.024	.579	.953
IL-12	.474	.119	.828	.242	.707
IL-13	.689	.108	.109	.478	.900
IL-15	.535	.121	.765	.298	.772
IL-21	.096	.059	.001	.000	.212
CCL-2	.109	.069	.001	.000	.244
CCL-4	.574	.121	.532	.336	.812
CCL-22	.545	.125	.703	.301	.789
CXCL-10	.420	.127	.497	.171	.669

a. Under the nonparametric assumption

b. Null hypothesis: true area = 0.5
